# Aerofoil optimization for improving the power performance of a vertical axis wind turbine using multiple streamtube model and genetic algorithm

**DOI:** 10.1098/rsos.180540

**Published:** 2018-07-25

**Authors:** Changping Liang, Huaxing Li

**Affiliations:** Aeronautics School, Northwestern Polytechnical University, Xi'an 710072, People's Republic of China

**Keywords:** vertical axis wind turbine, class and shape function transformation parametrization, genetic algorithm, aerofoil, optimization, multiple streamtube model

## Abstract

This paper reports on the optimization of the NACA0015 aerofoil for improving the power performance of a vertical axis wind turbine (VAWT). The target range of the chord *Re* is 3 × 10^5^–10^6^, the tip speed ratio (TSR) is 2–6 and the solidity is 0.2–0.6. This aerofoil is widely applied in small-scale VAWTs. In the optimization process, in which the class and shape function transformation parametrization method was used to perturb the aerofoil geometry, the thickness and camber of the aerofoil were selected as the constraints and the value of the maximum tangential force coefficient was chosen as the objective function. The aerodynamic performance of the aerofoil was calculated by combining the XFOIL program and Viterna–Corrigan post-stall model, while the aerofoil's performance was validated with computational fluid dynamic simulations. The results illustrated that, compared to an unoptimized NACA0015 aerofoil, the optimized aerofoil's lift to drag ratio was improved over a wide range of attack angles and the stall performance was gentler. The maximum lift coefficient, the maximum lift to drag ratio and the maximum tangential force coefficient were increased by 7.5%, 9% and 8.87%, respectively. Finally, this paper predicted the rotor efficiency with both the unoptimized and optimized NACA0015 aerofoils for different TSRs and different solidities using the multiple streamtube model. The results showed that the rotor with the optimized aerofoil has a higher efficiency.

## 1. Introduction

Energy is arguably the foundation of economic and social development and is closely related to human life and living environments. Wind power is a source of clean and renewable energy, and its technical applications are becoming increasingly mature. The vertical axis wind turbine (VAWT) has many advantages: such as omnidirectionality, low vibrations, low sound emissions, high safety factor, simple structure, ease of installation and convenience of control and repair. This has led it to become an enthusiastically studied device. There are many operational and geometric parameters that drive the performance of the VAWT, such as the solidity, blade numbers, pitch angle, tip speed ratio (TSR), Reynolds number, turbine shaft and aerofoils (table 1) [1–4]. Wind turbines rely on blades to draw wind energy, thus the aerodynamic performance of the aerofoils directly affects the utilization of the wind energy by a wind turbine. Sheldahl & Klimas [5] investigated the aerodynamic performance of the NACA0012, NACA0015, NACA0018 and NACA0021 aerofoils used in VAWTs. Also, Sheldahl & Klimas studied NACA four-series aerofoils typically used in VAWTs, notably the NACA0012, NACA0015 and NACA0018. However, Claessens [6] reported that common NACA0015 and NACA0018 symmetric aerofoils were not entirely suitable for VAWTs, and submitted that the original aerofoils must be improved. The NACA0018 aerofoil was improved by Claessens, who increased the thickness by 2% and the camber by 0.8% to produce the DU 06-W-200 laminar flow aerofoil. Under a negative angle of attack, the aerodynamic performances of the DU 06-W-200 and NACA0018 aerofoils were reasonable. Under a positive angle of attack, the DU 06-W-200 aerofoil had a higher coefficient of lift maximum and a wide range of low drag. Researchers modified the NACA0012 aerofoil and showed that increasing the aerofoil's camber improved the stall characteristics [7]. Liu *et al.* [8] found that appropriately thickening the aerofoil's trailing edge had little effect on the aerodynamic performance of the wind turbine, while the output power was enhanced slightly by thickening of the aerofoil. Ismail & Vijayaraghavan [9] modified the NACA0015 aerofoil used in VAWTs and investigated the effect of profile modifications. Simão & Geurts [10] demonstrated that optimizations based on the lift slope were the correct objective function for improving the VAWT power performance. In order to improve the performance of wind turbines, some previous studies on aerofoil optimization focused on optimizing the ratios of the lift to drag coefficients [11,12], but the effect of optimization is not obvious.

**Table 1.** Nomenclature.

*y*_TEU_	*y-*coordinates of the upper surfaces trailing edge
*y*_TEL_	*y-*coordinates of the lower surfaces trailing edge
*S*(*x*)	shape function
*Re*	Reynolds number
*C*_l_	lift coefficient
*C*_d_	drag coefficient
*C*_ls_	lift coefficient of stall
*C*_ds_	drag coefficient of stall
*α*	angle of attack
*α*_s_	stall angle of attack
*μ*	aspect ratio
*t*	aerofoil thickness
*c*	aerofoil camber
*Ma*	Mach number
*θ*	azimuthal angle
*V*_n_	normal velocity
*V*_c_	tangential velocity
*λ*	tip speed ratio
*C*_t_	tangential force coefficient
*N*	number of blades
*D*	diameter
*H*	height
*A*	swept area
*σ*	solidity
*c*	aerofoil chord
*U*_∞_	freestream velocity
*ω*	rotational speed

Because the tangential force is responsible for the power produced by a VAWT, we maximized the tangential force coefficient, and this study proposes an optimization of the NACA0015 aerofoil based on the class and shape function transformation (CST) parametrization and the non-dominated sorting genetic algorithm (NSGA-II). The target range of the solidity is 0.2–0.6, the chord *Re* is 3 × 10^5^–10^6^ and the TSR is 2–6. Owing to the fact that the angle of attack of the blades in a VAWT changes continuously, we propose maximizing the tangential force coefficient at seven angles of attack instead of maximizing the lift coefficient or ratio of the lift to drag coefficient at a single angle of attack. Additionally, this paper used the NSGA-II, the convergence of which is closer to the actual Pareto optimal level and the deviation is smaller. Therefore, compared to the previous methods, the optimization method proposed in this paper takes the fact that the angle of attack of the blades in a VAWT changes continuously into account, and proposes maximizing the tangential force coefficient at seven angles of attack instead of just maximizing the lift or ratio of the lift to drag coefficient at a single angle of attack, and this optimization method is more suitable for the optimization of the NACA0015 aerofoil which is widely applied in small-scale VAWTs. Compared to Zhang *et al*.'s [13] study of the optimization of a horizontal axis wind turbine (HAWT) aerofoil and Qu *et al*.'s [14] study of the optimal design of a VAWT aerofoil based on the complex optimum method, the aerofoil optimization method proposed in this paper gave better results. The optimized aerofoil exhibited better aerodynamic performance and the stall performance was gentler. The optimized aerofoil had a widely variable condition performance and its rotor showed a higher efficiency, and in addition to the improvement of the power performance, the thickness of the aerofoil is increased which is helpful for the structure of the VAWT, and thus the optimized aerofoil was more suitable for the operating conditions of a VAWT.

In recent years, some researchers have applied search algorithms in new ways. Zhang *et al*. [15] designed an improved transform coding algorithm for the implementation of a location-aware encoding strategy and Wei *et al*. [16] designed a fast and compact key frame search algorithm to achieve an efficient wide area localization system. The algorithm developed in this paper provides better search accuracy while having a convergence closer to the actual Pareto optimal level; thus, it can also be used as a localization and tracking method for wide area mobile AR applications.

## 2. Optimization method and the optimized aerofoil

### 2.1. Class and shape function transformation parametrization method

During the aerofoil optimization process, the aerofoil's geometric shape must be perturbed before optimization. In order to ensure that the perturbed points are smoothly and continuously connected, the mathematical parametrization method of the perturbation is used. The merits of the parametrization method have a very significant impact on the final optimization results, and therefore it is the key factor for determining the efficiency and results of the optimization. The parametrization method used in our optimization is a conventional method based on the CST proposed by Kulfan of the Boeing Company (Seattle, WA, USA). The CST parametrization method has clear geometric meaning, fewer control parameters, good adaptability and high accuracy. Kulfan noted that the method can be used for the parametrization of the aerofoil coordinates and that of other geometric features: such as the camber and arc [17]. The CST parametrization method has good potential for aerodynamic optimization of two-dimensional (2D) aerofoils and three-dimensional (3D) wings; thus it has also been widely applied to aerofoil optimization of wind turbines [18].

The Bernstein polynomials are used as the shape function of CST parametrization method, which is typically expressed as

2.1 $$y(x)=\sqrt{x}\cdot (1-x)\cdot \sum_{i=0}^{N}A_{i}\cdot x^{i}+x\cdot \Delta y.$$

For aerofoil, the expressions of geometric perturbation parametrization of the upper and lower surfaces are given as

2.2 $$y_{u}=C(x)\cdot S_{u}(x)+x\cdot y_{\mathrm{TEU}}$$

and

2.3 $$y_{l}=C(x)\cdot S_{l}(x)+x\cdot y_{\mathrm{TEL}},$$

where the subscripts u and l denote the upper and lower surfaces, respectively, *y*_TEU_ and *y*_TEL_ denote the *y-*coordinates of the upper and lower surfaces trailing edge. *C*(*x*) is the class function, which is used to limit the aerofoil type. Different types of aerofoils can be obtained by adjusting the value of *N*1, *N*2, where the class function is defined as

2.4 $$C(x)=x^{N1}\times {\left(1-x\right)}^{N2}.$$

For the round leading and sharp trailing aerofoil, the values of *N*1 and *N*2 are adopted as 0.5 and 1.0, respectively. *S*(*x*) is the shape function used to modify the basic shape formed by the class function; it is given as

2.5 $$S_{u}(x)=\sum_{i=0}^{N}A_{u_{i}}\times S_{i}(x)$$

and

2.6 $$S_{l}(x)=\sum_{i=0}^{N}A_{l_{i}}\times S_{i}(x),$$

where *A*_u*i*_ and *A*_l*i*_ are the coefficients to be determined. The aerofoil shape can be determined through determining the coefficients with least-squares fitting.

### 2.2. Genetic algorithm based on non-dominated sorting genetic algorithm

A genetic algorithm is a semi-random search optimization algorithm based on the Darwinian ‘survival of the fittest’ principle, and its specific mechanisms comprise the selection, crossover and mutation operations. A genetic algorithm based on non-dominated sorting was proposed by Srinivas and Deb that has a better distribution than the PAES and SPEA algorithms (as two multi-objective genetic algorithms based on an elitist strategy, these two algorithms have the advantage of creating a variety of Pareto optimal levels), and its convergence is closer to the actual Pareto optimal level [19]. The comparison was made between the three algorithms under the same test functions, and the test functions were given as

2.7 $$\begin{array}{rl} & \text{MOP}1:\;f_{1}(x)=1-\exp\left[-\sum_{i=1}^{3}{\left(x_{i}-\frac{1}{\sqrt{3}}\right)}^{2}\right], \end{array}$$

2.8 $$\begin{array}{rl} & f_{2}(x)=1-\exp\left[-\sum_{i=1}^{3}{\left(x_{i}+\frac{1}{\sqrt{3}}\right)}^{2}\right]\;-4\leq x_{1},x_{2},x_{3}\leq 4, \end{array}$$

2.9 $$\begin{array}{rl} & \text{MOP}2:\;f_{1}(x)=[1+{\left(A_{1}-B_{1}\right)}^{2}+{\left(A_{2}-B_{2}\right)}^{2}], \end{array}$$

2.10 $$\begin{array}{rl} & f_{2}(x)=[{\left(x+3\right)}^{2}+{\left(y+1\right)}^{2}], \end{array}$$

where *A*_1_ = 0.5 sin 1 − 2 cos 1 + sin 2 − 1.5 cos 2, *A*_2_ = 1.5 sin 1 − cos 1 + 2 sin 2 − 0.5 cos 2, *B*_1_ = 0.5 sin *x* − 2 cos *x* + sin *y* − 1.5cos*y*, *B*_2_ = 1.5 sin *x* − cos*x* + 2 sin *y* − 0.5 cos *y*, and

2.11 $$\begin{array}{rl} \text{MOP}3:\;f_{1}(x) & \;=\sum_{i=1}^{n-1}\left[-10\exp\left(-0.2\sqrt{\left(x_{i}^{2}+x_{i+1}^{2}\right)}\right)\right], \end{array}$$

2.12 $$\begin{array}{rl} f_{2}(x) & \;=\sum_{i=1}^{n}\left({\left|x_{i}\right|}^{0.8}+5\sin{\left(x_{i}\right)}^{3}\right). \end{array}$$

Comparing the real Pareto optimal boundary distance and its standard deviation of three algorithms, the result was calculated as table 2. It can be seen from table 2 that the convergence of the NSGA-II is closer to the actual Pareto optimal level and the deviation is smaller.

**Table 2.** The real Pareto optimal boundary distance and its standard deviation.

algorithm	MOP1		MOP2		MOP3	
NSGA-II	0.361	0.00068	0.445	0.00043	0.387	0.00164
PAES	1.609	0.00671	1.341	0.00495	1.087	0.00687
SPEA	0.740	0.00748	0.880	0.00508	0.733	0.00175

### 2.3. Calculation of the aerofoil's aerodynamic performance

The calculation of the aerodynamic performance of an aerofoil is the critical step in aerofoil optimization design. For small VAWTs, which usually work under a low Reynolds number between *Re* = 0.5 ×10^5^ and 3 × 10^5^, the adopted aerofoil is very sensitive to changes in the strength of turbulence, the roughness of the aerofoil surface, its self-vibration, etc. XFOIL is a viscous and non-viscous iteration program that is widely used to analyse the aerodynamic performance of aerofoils with low Reynolds numbers. It can not only solve the nonlinear coupling of the single bubble that occurs among the viscous, transition and non-viscous formulas, but can also illustrate complex physical phenomena such as transition bubbles, etc. The performance of the NACA63–615 and other aerofoils was calculated using XFOIL and by wind-tunnel experiments, and the results showed that the data obtained by both methods were in good agreement before stalling [10]. When using XFOIL to calculate the performance, first load the aerofoil's data, set the Reynolds number and Mach number and then calculate the lift and drag coefficients at the corresponding angle of attack. When aerofoils are in stall conditions, the aerodynamic performance is similar to that of flat disturbed flow with a high angle of attack and no longer depends on the aerofoil's geometry. Therefore, the Viterna–Corrigan post-stall mode was used for calculating the aerodynamic performance after aerofoil stalling [20]. The literature [20] showed that the data calculated with the Viterna–Corrigan post-stall model had a good fit with the experimental data. The Viterna–Corrigan post-stall mode is specifically described as follows:

2.13 $$C_{l}=\frac{1.11+0.018\mu }{2}\sin2\alpha +\left[C_{\mathrm{ls}}-(1.11+0.018\mu )\sin\alpha_{s}\cos\alpha_{s}\right]\frac{\sin\alpha_{s}\cos^{2}\alpha }{\cos^{2}\alpha_{s}\sin\alpha }$$

and

2.14 $$C_{d}=(1.11+0.018\mu )\sin^{2}\alpha +\left[\frac{C_{\mathrm{ds}}}{\cos\alpha_{s}}-(1.11+0.018\mu )\sin^{2}\alpha_{s}\right]\cos\alpha ,$$

where *C*_l_ and *C*_d_ are the lift and drag coefficients, *C*_ls_ and *C*_ds_ the lift and drag coefficients of stall, *α* and *α*_s_ the angle of attack and stall angle of attack and *μ* the aspect ratio.

### 2.4. Aerofoil optimization design

#### 2.4.1. Design variables and constraints

During aerofoil optimization design, the selection of design variables has a significant impact on the optimization process. The thickness and camber of the aerofoil are very important geometric parameters. When the Reynolds numbers are low, the lift coefficient, drag coefficient and stall angle can be increased by increasing the camber of the aerofoil, while the sensitivity of the aerofoil to the leading edge roughness can be reduced concurrently. Appropriately increasing the aerofoil thickness can increase the starting torque of a VAWT, enhance the structural strength of the blade and improve the aerodynamic performance of the aerofoil. For a VAWT aerofoil, Claessens [6] showed that an aerofoil with a 15–18% thickness relative to the chord length and a camber that did not exceed a value of 6% had better aerodynamic performance. Therefore, the key design variables were identified as the aerofoil thickness *t* and camber *c*, with constraints of 15% ≤ *t* ≤ 18% and 0% ≤ *c* ≤ 6% with respect to the chord length. The Reynolds number used was 300 000, while the Mach number was 0.032 and multi-point optimization was executed for an NACA0015 aerofoil at seven angles of attack.

#### 2.4.2. Determination of the objective function

**Figure 1.**
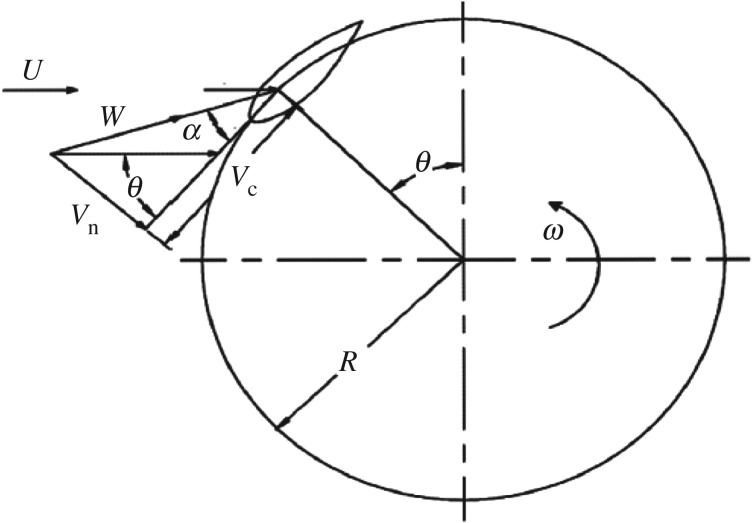
Force analysis of a VAWT.

As figure 1 shows, *θ* is the azimuthal angle, *ω* is the rotational angular velocity, *V*_c_ is the tangential velocity, *V*_n_ is the normal velocity and *α* is the angle of attack. With the rotation of the VAWT, the wind relative to the VAWT blades has a change both in tangential and normal. As a result, *V*_c_ and *V*_n_ can be expressed with the induced velocity (*U*) and TSR. *W* is the effective wind velocity which can be written in terms of *V*_c_ and *V*_n_.

2.15 $$\begin{array}{rl} V_{c} & \;=U(\lambda +\cos\theta ), \end{array}$$

2.16 $$\begin{array}{rl} V_{n} & \;=U\sin\theta , \end{array}$$

2.17 $$\begin{array}{rl} W & \;=\sqrt{V_{c}^{2}+V_{n}^{2}} \end{array}$$

2.18 $$\begin{array}{r} \text{and}\;\alpha =\tan^{-1}\left(\frac{V_{n}}{V_{c}}\right), \end{array}$$

where *λ* = *ωR/U_∞_* is the TSR.

During the aerofoil's optimization design process, optimization of the lift coefficient, drag coefficient and lift to drag ratio along with other parameters can be chosen as the primary goals of optimization, with the overall goal of the optimization process being to select the parameters to achieve the optimal solutions. For a VAWT, the tangential force coefficient *C*_t_ is always selected to evaluate the aerodynamic performance of a VAWT aerofoil at rated operational levels. Therefore, the objective function is determined as the maximum *C*_t_ that can be expressed with *C*_l_ and *C*_d_:

2.19 $$C_{t}=C_{l}\sin\alpha -C_{d}\cos\alpha .$$

#### 2.4.3. Establishment of the optimization process

**Figure 2.**
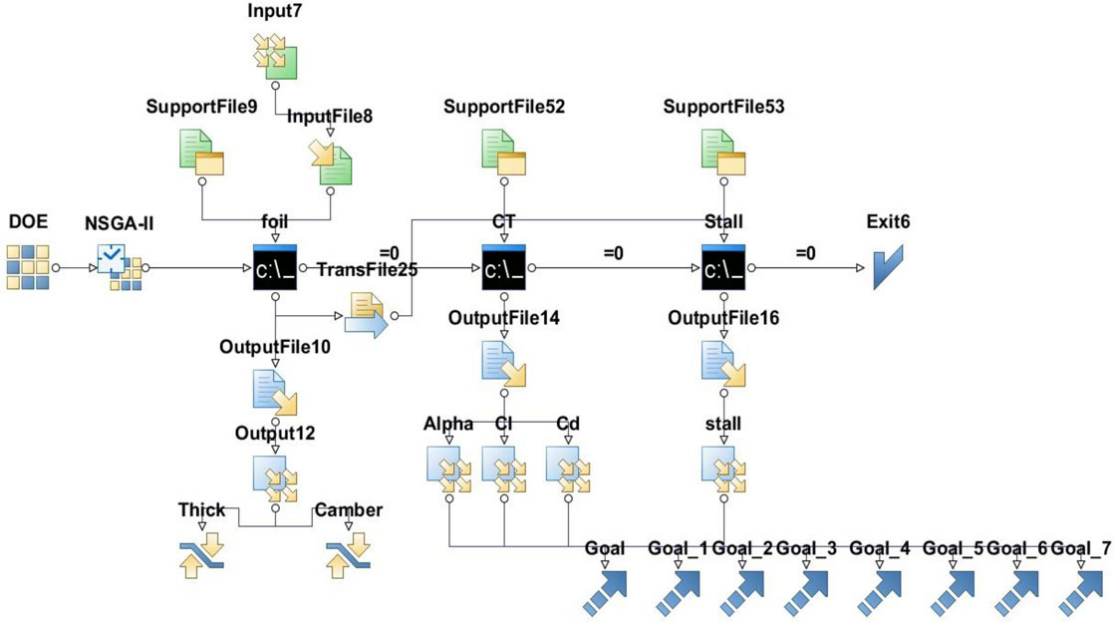
The optimization process used by modeFRONTIER.

ModeFRONTIER is a general multi-objective and multi-disciplinary optimization software developed by the Italian company ESTECO. It establishes the corresponding optimization process based on the differences in the actual optimization problems. The entire workflow includes the data flow and logic flow. The data flow is the process to get the output variables, while the input variables are imported through the data interface of the integrated module. After the calculations are completed, the data are exported to the next integrated module to resume more calculations. The logic flow is the process to generate the input variables, control the optimization process and assess the output variables. The decision variables, constraints and objective functions of the optimal mathematical model correspond to the input variables, constraints and output variables of the workflow [21]. The aerofoil optimization process is shown in figure 2.

### 2.5. Shape of the optimized aerofoil

**Figure 3.**
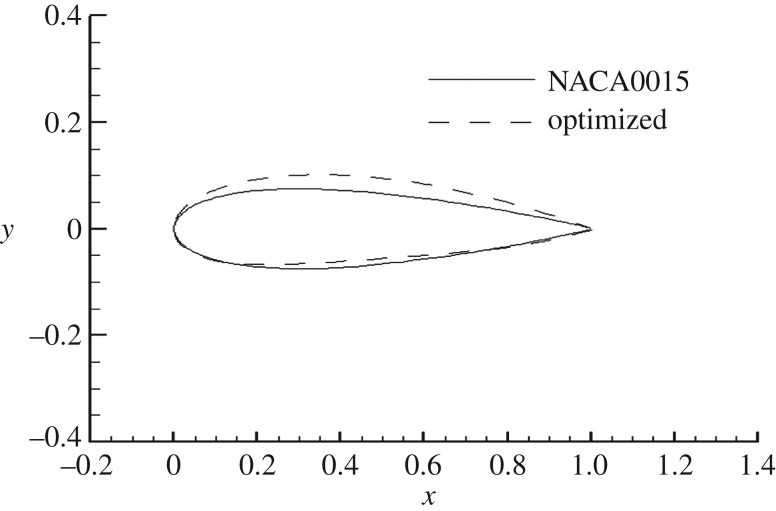
Comparison of aerofoil geometry.

The optimized aerofoil was found to have a thickness of 16.65% and a camber of 2% relative to the chord length. The shapes of the optimized and unoptimized NACA0015 aerofoils are shown in figure 3. The geometric shape of the optimized aerofoil shows changes to the thickness, camber and leading edge radius as they were all increased by varying degrees.

## 3. Computational fluid dynamic numerical simulation and multiple streamtube model

### 3.1. Governing equations and boundary conditions

In the computational fluid dynamic (CFD) software Ansys Fluent, aerofoils are simulated with the Reynolds-averaged Navier–Stokes (RANS) equation [22].

**Figure 4.**
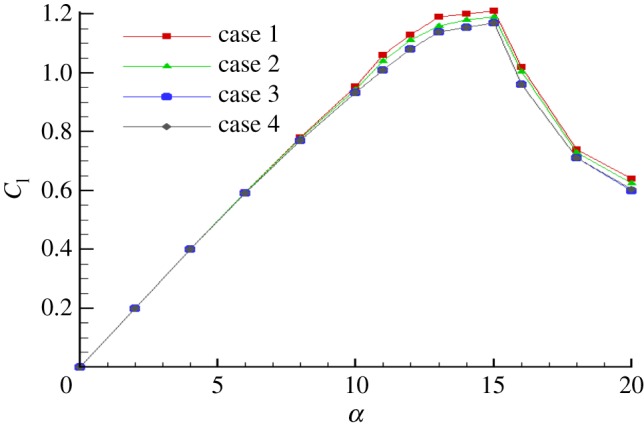
Grid independence study.

**Figure 5.**
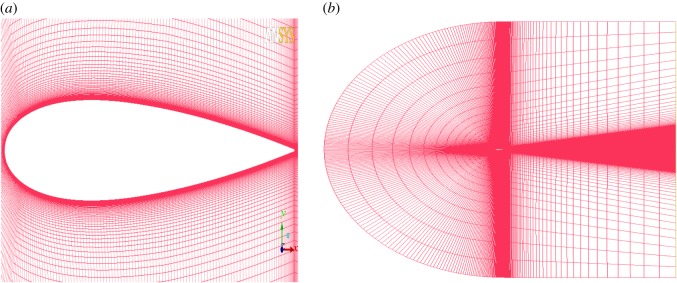
Computational and aerofoil magnified view grids. (*a*) Aerofoil magnified view grid and (*b*) computational grid.

The aerofoil has a chord length of *c* = 0.4 m, a long enough domain (50*c*) that is chosen to preclude the effect of the outlet boundary condition. The grid independence study is shown in table 3; figure 4 shows the lift coefficient simulated by different grids. The grid of case 3 reaches the requirement of grid independence, and the results given later in this article are based on grid case 3. According to the grid-convergence index of Roache [23], the error is approximately 5% when using grid case 3. The aerofoil is meshed with the *C*-type grid with 800 nodes over the aerofoil surface. The first layer of the wall thickness is 0.00001*C* to make sure the value of *y*+ is less than 1. The mesh is shown in figure 5.

**Table 3.** The grid independence study.

grid	${Y^{+}}_{\max}$	grid number
case 1	4	58 600
case 2	2	73 800
case 3	1	98 460
case 4	0.9	110 400

A constant velocity was set at the inlet boundary and the initial gauge pressure was 0 Pa, while the constant pressure of 1 atm was specified at the outlet. The aerofoil's walls were set as the no-slip wall condition and the pressure corrected according to the flow field values computed by the RANS equation, while the other boundaries were set as symmetric boundary conditions. Discretization has been preceded by the finite-volume method with a second-order upwind scheme for all variables; the turbulent intensity and turbulent viscosity ratio are both 0.01. To obtain a more appropriate turbulence model for the investigation of the aerofoil flow field structure, the *k–ω* shear stress transport (*k–ω* SST) and Spallart–Almaras (SA) models were used to calculate the lift coefficients. The lift coefficients tested by the two turbulence models were then compared to the experimental data. The aerofoil was analysed at 12 different angles of attack ranging from 0° to 20°, at the chordal Reynolds number of 3 × 10^5^.

**Figure 6.**
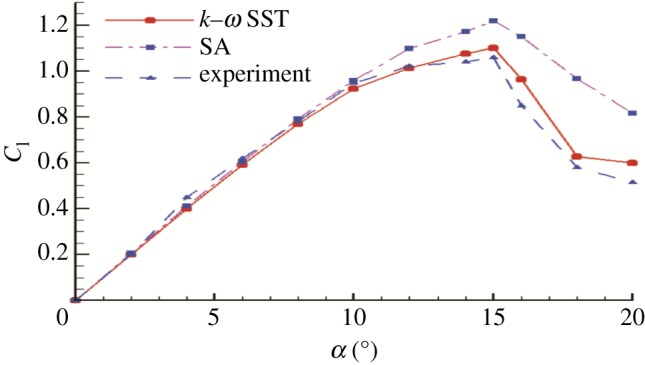
Comparison of CFD and experimental lift coefficients of NACA0015 aerofoil.

It can be seen from figure 6 that the *k–ω* SST turbulence model has a closer prediction of lift coefficient than the SA turbulence model; hence, it is considered the better model. The turbulent intensity and the turbulent viscosity ratio were both 1%.

### 3.2. Multiple streamtube model

In order to analyse the aerodynamic performance of the VAWT, the free wake model, rigid wake model and streamtube model have been built up. The free wake model has been successfully applied to propeller and aerofoil design, while owing to its complexity and the calculation requiring a lot of computing time, it is unsuitable to predict the performance of a VAWT. Each streamtube has its own speed, and the speed change in each streamtube is decided by parallel orientation with the free stream. Multiple streamtubes model was firstly proposed by Strickland in 1975. The calculation process of the multiple streamtubes model is relatively simple and has a higher accuracy than the single streamtube [24].

**Figure 7.**
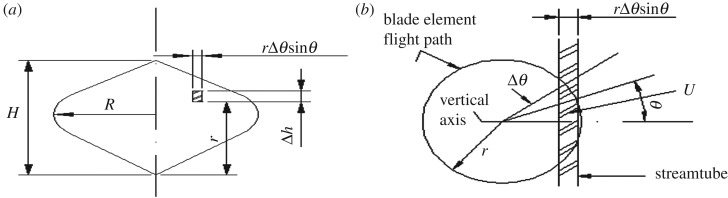
Typical streamtube. (*a*) Upstream view and (*b*) plane view.

Suppose some identical streamtubes through the rotor, each streamtube streamwise momentum equation makes the force acting on the aerofoil of the blade equal. In figure 7 is a typical streamtube, where Δ*h* is the vertical height, *rΔθ*sin*θ* is the width, the local radius is *r* and the rotor phase angle is *θ*. The velocity after passing through the streamtube is denoted by *V*.

**Figure 8.**
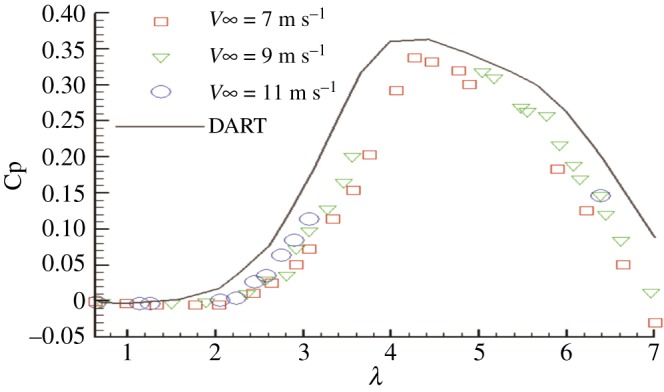
Comparison of DART with Sandia test data.

The computer program code associated with the Strickland multiple streamtube model known as DART is used to calculate the performance of small wind turbine. When using DART to calculate the performance, first load the tangential force and normal force coefficient of aerofoil, set the Reynolds number, Mach number and solidity, and then calculate the performance of the turbine. Wind-tunnel tests of two 2 m diameter rotors were conducted in the LTV wind tunnel in May 1975. A rotor with three blades was tested, *NC/R* = 0.27, and the rotor blade aerofoil was the NACA0012 aerofoil. The free stream velocities of the test were 7, 9 and 11 m s^−1^. For the 9 m s^−1^ wind speed, the Reynolds numbers ranged from 0.10 × 10^6^ to 0.36 × 10^6^. Data used in the DART model were from Jacobs & Sherman [25]. The comparison between DART and test results for a blade (Reynolds number 0.3 × 10^6^) is shown in figure 8.

Figure 8 shows relatively good agreement of the power coefficients between the DART and Sandia test data for a blade with a Reynolds number of 0.3 × 10^6^. The failure of the curve to agree exactly was partially due to the difference in Reynolds numbers between the DART and Sandia test data.

### 3.3. Unsteady Reynolds-averaged Navier–Stokes computational fluid dynamic simulations

**Figure 9.**
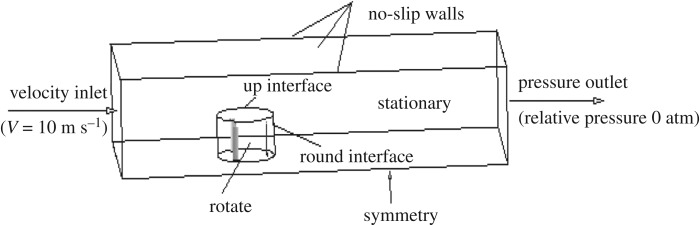
Boundary conditions.

To judge whether the optimized aerofoil really improves the power performance of a VAWT, a 3D transient URANS CFD simulation for a rotating VAWT at several different TSRs *λ* with the original NACA0015 and the optimized aerofoil (OPT) is used to evaluate the aerodynamic performance of the VAWT. The computational settings are from the literature [26,27]. The computational domain of the 3D physical model is 9*D* × 4*D* × 2.5*D*. The distances between the entrance, exit and both sides of the boundary from the centre of rotation of the rotor are 3*D*, 6*D* and 2*D*, respectively. The computational domain consists of a stationary domain and a rotation domain, and the two domains are coupled with a slip grid. The discrete method was the finite-volume method and the turbulence model was *k–ω* SST. The boundary conditions were set as shown in figure 9. The geometric and operational characteristics of the wind turbine are shown in table 4.

**Table 4.** The geometric and operational characteristics of the wind turbine.

characteristics	turbine
number of blades, *N*	3
diameter, *D* (m)	2.5
height, *H* (m)	3
swept area, *A* (m^2^)	7.5
solidity, *σ*	0.48
aerofoil chord, *c* (m)	0.4
tip speed ratio, *λ*	0.5–3
freestream velocity, *U*_∞_ (m s^−1^)	10
rotational speed, *ω* (rad s^−1^)	4–24

## 4. Results and discussion

Numerical simulations of the flow characteristics of an NACA0015 aerofoil at a wind speed of 11 m s^−1^ were carried out at different angles of attack using the *k–ω* SST model. The simulation results were analysed using the post-CFD software Tecplot 360 (Tecplot, Inc., Bellevue, WA, USA).

**Figure 10.**
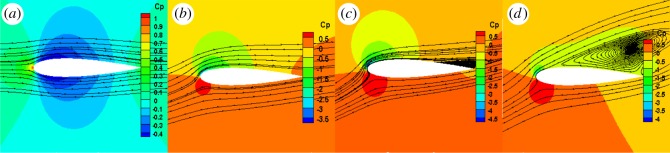
Flow streamlines and pressure contours of NACA0015 aerofoil. (*a*) 0°, (*b*) 10°, (*c*) 15° and (*d*) 20°.

**Figure 11.**
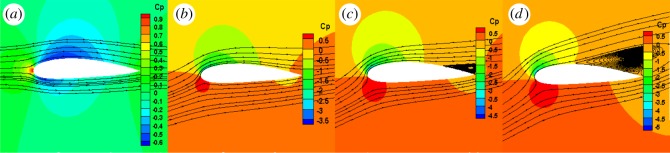
Flow streamlines and pressure contours of OPT aerofoil. (*a*) 0°, (*b*) 10°, (*c*) 15° and (*d*) 20°.

**Figure 12.**
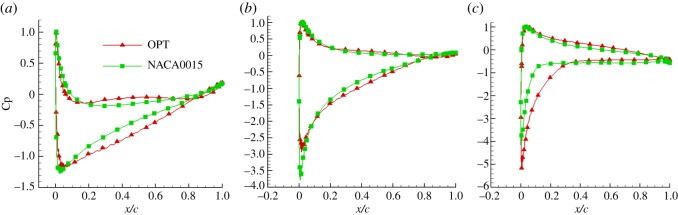
Pressure coefficient plots. (*a*) 4°, (*b*) 10° and (*c*) 20°.

As figures 10–12 show, the leading edge experienced high pressure, while the trailing edge experienced low pressure. The larger the angle of attack, the greater the difference in pressure was between the upper and lower surfaces. When the attack angle was zero, the pressure on the upper and lower surfaces of the NACA0015 aerofoil was symmetrical, thus there was no lift. When the pressure of the optimized aerofoil's lower surface was larger than that of the upper surface, the optimized aerofoil had lift. The pressure coefficient plots showed that the leading edge experienced high pressure, while the trailing edge experienced low pressure. The pressure distribution on the aerofoil's lower surface showed positive values which produced positive lift force. The upper surface showed positive lift values, which produced negative lift force. Owing to the optimized aerofoil curvature of the leading edge and the camber of the trailing edge being increased, the pressure difference on the optimized aerofoil's upper and lower surfaces was larger than that on the NACA0015 aerofoil, thus the lift coefficient of the optimized aerofoil was greater than that of the NACA0015 aerofoil. In the post-median of the aerofoil, the pressure difference on the optimized aerofoil's upper and lower surfaces exhibited a smooth transition, indicating that the load pressure gradient was uniformly reduced from the aerofoil's centre to the trailing edge. This results in the aerofoil having good mechanical performance. In addition, the pressure gradient on the optimized aerofoil's upper surface, near the leading edge, changed more gently. When the angle of attack was greater than 15°, flow separation appeared at the trailing edge of the two aerofoils, and an intensity separation vortex formed. When the angle of attack was 20°, the flow separation phenomenon was obvious. However, compared to the NACA0015 aerofoil, the separation point of the optimized aerofoil was opposite and the separation area decreased. This is because when the curvature of the optimized aerofoil leading edge was larger, the air flow accelerated quickly to curb the premature separation of the flow which made the separation point opposite.

**Figure 13.**
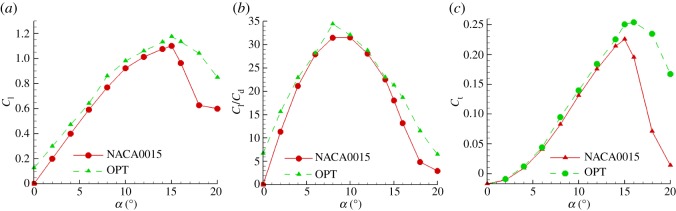
Comparison of (*a*) lift coefficients, (*b*) lift–drag ratios and (*c*) tangential force coefficient.

Figure 13 shows that the lift coefficients, lift to drag ratios and tangential force coefficients of the optimized aerofoil were higher than those of the NACA0015 aerofoil. The maximum lift coefficient of the two aerofoils was achieved at an attack angle of 15°, but the maximum lift coefficient of the optimized aerofoil was 1.175. This was 7.5% greater than that of the NACA0015 aerofoil. When the angle of attack was greater than 15°, the lift coefficient of the two aerofoils began to decrease. This occurred because with the increasing angle of attack, the flow separation occurring in the aerofoil's trailing edge resulted in a separation vortex, and the breakdown of the separation vortex made the circulation of the aerofoil decrease. However, the lift coefficient of the optimized aerofoil was reduced to a lesser extent than that of the NACA0015 aerofoil which dropped dramatically. This indicated that the optimized aerofoil stalled more gently, further demonstrating that the stall performance of the optimized aerofoil has been improved compared to the NACA0015 aerofoil. The maximum lift to drag ratio of the optimized aerofoil was achieved at a 13° angle of attack, its maximum lift to drag ratio increased by 9% and its maximum tangential force coefficient increased by 8.87%. When the aerofoil stalled, the tangential force coefficient of the NACA0015 aerofoil dropped dramatically, while that of the optimized aerofoil decreased more slowly.

**Figure 14.**
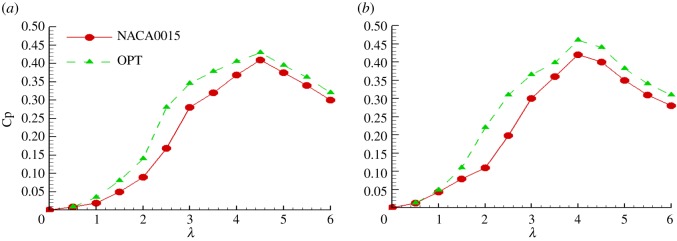
Comparison of the NACA0015 and optimized aerofoils. (*a*) *NC/R* = 0.18 and (*b*) *NC/R* = 0.27.

Figure 14 shows the performances of a two-bladed rotor with a value of *NC/R* = 0.18 and a three-bladed rotor with a value of *NC/R* = 0.27 that were predicted using the multiple streamtube model, in which the blade Reynolds number was 3 × 10^5^. For different TSRs and different solidities, the rotor efficiency with the optimized aerofoil was higher than that with the NACA0015 aerofoil. When the TSR was less than 1, the effect was not obvious. However, when the TSR was greater than 1, the optimized aerofoil's rotor efficiency was significantly higher than that of the NACA0015 aerofoil. Moreover, when the TSR was between 2 and 4, the efficiency increased considerably, but when the TSR was greater than 4, the increase in efficiency was reduced. When the value of *NC/R* was 0.18, the maximum efficiency of the rotor was achieved at a TSR of 4.5, while at an *NC/R* value of 0.27, the maximum efficiency of the rotor was achieved at a TSR of 4. The increase in the highest efficiency point was 4.88% and 9.5%, respectively. Thus, the rotor with the optimized aerofoil had a higher efficiency, and was more suitable for VAWT operations.

**Figure 15.**
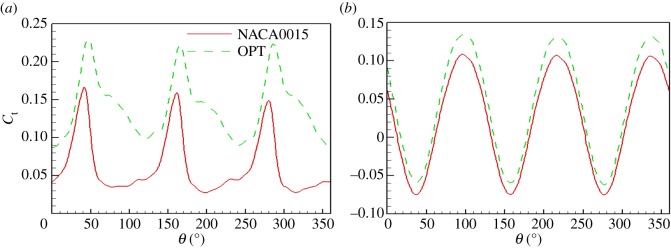
Torque coefficients of the turbine. (*a*) *λ* = 0.5 and (*b*) *λ* = 3.

**Figure 16.**
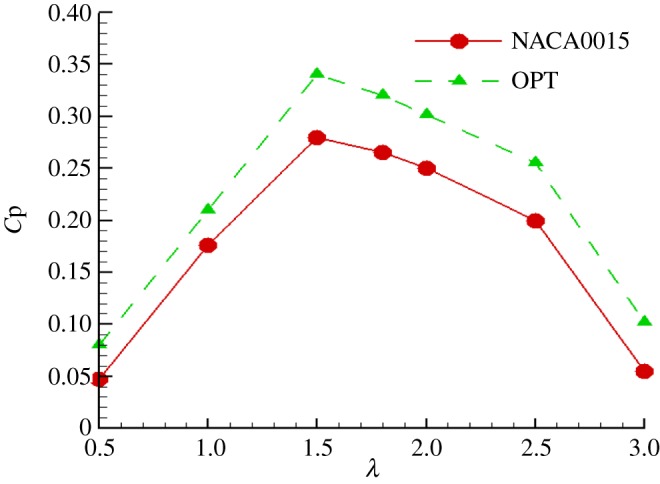
Power coefficients of the turbine under different TSRs.

Figure 15 shows the instantaneous torque coefficient change with azimuth angle. It can be seen that under different TSRs, the instantaneous torque coefficients with azimuth angle have a typical periodicity. In one rotation cycle of the rotor, three peaks and troughs appear in the torque coefficient, but the azimuth angles of the peaks are different under different TSRs. At the TSR of 0.5, the three peaks of the torque coefficient appear at azimuth angles of 40°, 160° and 280°; at the TSR of 3, the three peaks appear at azimuth angles of 90°, 210° and 330°, respectively. Compared with the original NACA0015 aerofoil, the maximum and minimum instantaneous torque coefficients of the turbine with the optimized aerofoil have different degrees of improvement under different TSRs. It can be seen that the torque coefficient of the turbine with the optimized aerofoil is higher. Figure 16 shows the power coefficients of the turbine under different TSRs. It can be seen that when the TSR *λ* < 1.5, the power coefficient of the turbine increases with increasing TSR; when the TSR *λ* = 1.5, the power coefficient reaches the maximum; when the TSR *λ* > 1.5, the power coefficient decreases with increasing TSR. On the whole, under different TSRs, the power coefficient of the turbine with the optimized aerofoil is higher than that of the turbine with the NACA0015 aerofoil, and the power coefficient of the turbine is improved by 17.6% at the optimal TSR. This shows that the optimized aerofoil has obvious improvement effect on the aerodynamic performance of the turbine under different TSR conditions. The turbine with the optimized aerofoil has higher efficiency and a wider high efficiency zone.

## 5. Conclusion

This paper reports on the optimization of the NACA0015 aerofoil for improving the power performance of a VAWT; the target range of chord *Re* is 3 × 10^5^–10^6^, the solidity is 0.2–0.6 and the TSR is 2–6. In this paper, a process for optimizing the NACA0015 aerofoil to obtain a maximum *C_t_* leveraging CST and NSGA-II was proposed. CFD simulations were used to obtain the data needed after the optimization. The *k–ω* SST turbulence model was used in the CFD studies because it provided a more accurate prediction of the lift coefficient in both the pre-stall and post-stall regions.

The joint optimization approach was used to determine the aerofoil camber and thickness, and it was found that the thickness and camber of the optimized aerofoil exhibited an increase compared to the NACA0015 aerofoil, as did the camber and thickness of the leading edge. After the optimization, the differential pressures of the upper and lower surfaces increased, the lift performance improved and the maximum lift coefficient increased by 7.5%.

Owing to the multi-point nature of the optimization, the optimized aerofoil demonstrated good performances over various variable conditions. The lift to drag ratio was improved over a wide range of angles of attack, and the maximum lift to drag ratio increased by 9%. The stall angle of attack for both aerofoils was 15°. The optimized aerofoil had a separation point that was reversed and a decreased separation area. It also stalled more gently, and had an improved stall performance. After the aerofoil stalled, the lift coefficient, lift to drag ratio and tangential force coefficient of the optimized aerofoil were much higher than those of the NACA0015 aerofoil; therefore, the optimized aerofoil was more suitable for VAWT operations.

Moreover, this paper verified the accuracy of the multiple streamtube model and predicted the rotor efficiencies of the optimized and NACA0015 aerofoils for different TSRs and solidities. Finally, this paper performed a 3D transient URANS CFD simulations for a rotating VAWT at several different TSRs with the original NACA0015 and the optimized aerofoil to evaluate the aerodynamic performance of a VAWT. The result shows that under different TSRs, the power coefficient of the turbine with the optimized aerofoil is higher than that of the turbine with the NACA0015 aerofoil, and the power coefficient of the turbine is improved by 17.6% at the optimal TSR. Therefore, the rotor with the optimized aerofoil had a higher efficiency, and the optimized aerofoil is more suitable for the operating conditions of a VAWT. This proposed method could also be used to optimize the aerofoils of HAWTs and other propeller-based machines.

However, the limitations of this work are that the proposed method is currently only applicable to the optimization of 2D aerofoils. Further studies should be conducted to combine the processes used in this paper with the aerodynamic performance of VAWT blades. Additionally, this work does not study the effect of the optimized aerofoil on the self-starting of the turbine.

## Data accessibility

This article does not contain any additional data.
